# Phonetic Accommodation on the Segmental and the Suprasegmental Level of Speech in Native–Non-Native Collaborative Tasks

**DOI:** 10.1177/00238309211050094

**Published:** 2021-10-20

**Authors:** Christiane Ulbrich

**Affiliations:** University of Konstanz, Germany

**Keywords:** Phonetic accommodation, collaborative task, Spanish–German, segmentals, suprasegmentals

## Abstract

This paper presents the investigation and analysis of speech accommodation effects in data obtained from Spanish learners of German with varying proficiency levels. The production data were recorded during a collaborative map task of the Spanish learners of German among each other and with a native speaker of German. The map task was designed to target words and phrases with specific segmental and suprasegmental characteristics. These characteristics were derived from contrastive analyses of Spanish and German. The main objectives of the paper were to investigate whether segmental and suprasegmental characteristics of the target language German are affected by phonetic accommodation to varying degrees and whether these differences depend on the proficiency level of the speaker or the interlocutor. The statistical analysis, using regression analyses, revealed inconsistent accommodation effects across learners of different proficiency levels as well as different linguistic phenomena. In line with previous findings the results can best be accounted for by an adaptation of a dynamic system approach.

## 1 Introduction

Cross-linguistic influences in bilingual or multilingual contexts have been the focus of numerous studies. Early studies on learners acquiring a second language (L2) after first language (L1) maturation have primarily dealt with the impact of the L1 on the L2. More recently though the reverse, that is, the impact of L2 on the L1 has been included in the research agenda. The shift of the focus from one-directional to bi-directional cross-linguistic influences (or even omni-directional cross-linguistic influences considering third language or L1 contexts) results from the growing recognition of the moldability of linguistic representations. Insights into the nature of reshaping linguistic representations will allow for a more thorough understanding of linguistic development and the impact of specific measures and interventions.

Accommodation effects provide concrete evidence for the flexibility of linguistic representation. Accommodation effects have been demonstrated in various different communication settings, for example, in cross-dialectal communication (see Auer et al., 2005; Babel, 2009; Evans & Iverson, 2007; Hinskens & Auer, 2005; Macleod, 2012; Trudgill, 1986), in clinical communication (Winn, 2020), as a result of diachronic change (Evans, 2015; Höder, 2009), and in bilingual or multilingual communication (e.g., Burin & Ballier, 2007; Khattab, 2009; Kim & Bradlow, 2011; Lewandowski & Jilka, 2019; Tobin et al., 2017; Ulbrich, 2019).

Accommodation has also been described on all linguistic levels of spoken language (for semantics (and the interface with pragmatics) see e.g., Beaver & Zeevat, 2007; Heim, 1992; Katzir & Singh, 2013; Libermann, 2012; for multiple levels of linguistics see Ferrara, 1991; for syntax see Bock, 1986; Gries, 2005; for vocabulary see Boghrati, et al., 2016; Gueguen, 2009; Jacob et al., 2011; Van Baaren et al., 2003; for morphology see Dunstan, 2010; Trudgill, 1986; and for phonetics and phonology see Chang, 2013; Fabiano-Smith et al., 2010; Nilsenová & Swerts, 2012; Pardo, 2006; Pardo et al., 2013; Sancier & Fowler, 1997; Tobin et al., 2017). More recently, accommodation effects have also been demonstrated in text-based communication, more specifically in online-communication (e.g., Bunz & Campbell, 2004; Danescu-Niculescu-Mizil et al., 2011; Scissors et al., 2009). In addition to the analysis of various linguistic phenomena, scholars have investigated several factors influencing phonetic accommodation such as the interviewer’s/interlocutor’s accent or gender (e.g., Bilous & Krauss, 1988; Willemyns et al., 1997), or power structures (e.g., Lutzross et al., 2018; for a comprehensive overview see Kim & Bradlow, 2012). Other factors shown to influence accommodation in bilingual or multilingual conversations are talent (Lewandowski, 2012) and language experience (Llanos & Francis, 2017).

Already in the 1970s, with the proposal of communication accommodation theory (Giles, 1973), it was demonstrated that speakers consciously and unconsciously modify their speech to increase social compatibility, social approval and affiliation, integration and identification as also suggested in the similarity–attraction paradigm by Byrne (1971). Speakers, however, can also emphasize their distinctiveness in the ambition to distance themselves from an interlocutor (see for discussion Dragojevic et al., 2016) by either diverging from the interlocutor’s verbal and non-verbal behavior (Babel, 2009; Bourhis & Giles, 1977) or by maintaining their own (linguistic) identity (Bourhis, 1984). In bilingual or multilingual communication, highly proficient speakers may be able to opt for the interlocutor’s language to signal shared identity and social affiliation. They may also be able to adhere to their own language to distance themselves from respective outgroups or interlocutors and/or signal strong identification to their ingroup (Zuegler, 1991). Previous research, however, has revealed that the L2 also imposes extra demands on the L2 speakers and that this effect may block possible alignment processes (Kim & Bradlow, 2011).

As mentioned before, there is a long-standing research tradition on accommodation predominantly in sociolinguistics and it is a process found in very early “conversations” as evidenced in the convergence of manual and facial gestures in adult–infant interaction (Nagaoka et al., 2007). Nonetheless, the mechanisms underlying the process are still not very well understood.

Earlier works focused on the production and perception of converging (and diverging) speech patterns in relation to interlocutors’ attitudes depending on discourse–contextual, situational or social factors (Bourhis & Giles, 1977; Giles, 1973). Such initial attempts examined the evaluation of speakers’ competence and social attributes associated with them by listeners. More recent studies are specifically concerned with acoustic–phonetic properties of speech with a primary focus on the segmental level. This is surprising, since already in the 1980s Gumperz (1982) mentions the importance of compliance to cultural linguistic norms in communication, such as where to put emphasis and how to use intonation. Moreover, it is well known that prosodic characteristics of speech contribute to foreign accentedness (Ulbrich & Mennen, 2016) and can impair comprehensibility (Trofimovich & Baker, 2006). Nonetheless, the suprasegmental level of speech has rarely been considered in the scientific debate concerning speech accommodation in multilingual contexts. The present paper intends to add to some existing evidence that both segmental and prosodic characteristics can be adjusted in response to an interlocutor.

### 1.1 Mechanisms underlying speech accommodation

Two types of motives for accommodation are distinguished in the early works on accommodation: affective motives; and cognitive motives. The affective motives refer to the speaker’s intention to handle social proximity, to maintain and create positive personal and social identities (Tajfel & Turner, 1986) and to express attitudes in relation to the context, the topic and the interlocutor(s). Such attitudes, however, are affected by inadequate or insufficient language skills of non-native speakers (Stell & Dragojevic, 2017). Corbett (2019), for instance, showed that the realization of coda consonants /s/, /ɾ/, and /n/ of eight native speakers of New York Dominican Spanish varied depending on the proficiency level of the interlocutor. The direction of accommodation varied between highly proficient interlocutors and those with a low proficiency level on the one hand and interlocutors with mid-level proficiency on the other. While participants converged towards the former, they initially diverged from the latter. Corbett (2019) also reports variation in the choice of socially associated variants. In conversation with the highly proficient interlocutors, the native speakers used overtly prestigious variants, whereas mid-level proficient and native speakers used emblematic variants. The choice was interpreted as an intentional signal for outgroup and ingroup membership. It illustrates accommodation as socially motivated, voluntary and conscious alternations, hence as a dynamic process that speakers strategically apply to gain social approval (Gregory & Webster, 1996; Holtgraves, 2002). In addition to the affective goals to either evoke listeners’ social approval or to maintain social identities, Thakerar et al. (1982) added a cognitive goal, namely the attainment of communicative efficiency. This implies that accommodation was seen as a means to facilitate language processing and thereby to enhance communicative success (Shepard et al., 2001).

Another line of research proposes accommodation to be largely automatic. Trudgill (2008), for instance, argues that accommodation occurs automatically, providing the basis for social group identity rather than causing it. In line with this assumption, previous research demonstrated automatic accommodation effects unrelated to social factors but because of factors such as novelty, atypicality, recency and immediacy (Enzinna, 2018). Accommodation in that view is an involuntarily and unconscious phenomenon, as proposed by Garrod and Pickering (2007) and Pickering and Garrod (2006) in the interactive alignment account. Accommodation occurs because interaction between individuals is the basic form of language use whereby self-monitoring and repair mechanisms are employed to ensure a common ground between interlocutors. The process is automatic and “only depends on simple priming mechanisms” (Pickering & Garrod, 2004, p. 188). The assumption that accommodation is automatic and subconscious also finds support in studies of alignment in situations without any interpersonal contact rendering accommodation as a low-level mechanism of the perceptual-motor system (Dias & Rosenblum, 2016; Fowler et al., 2003; Mitterer & Müsseler, 2013). Such low-level mechanisms were observed in imitation and shadowing experiments, deliberately excluding social factors from the experimental set up. The evidence for automatic accommodation led to the proposal of an exemplar-based model of accommodation. Goldinger (1998), for instance, found fewer accommodation effects in high-frequency words as compared to low-frequency words. Phonetic accommodation was thus interpreted as an automatic reflex of the cognitive system. Moreover, the perception–production-link has been suggested to cause the automatic effect in that individuals memorize perceived speech gestures consequently causing their imitation (Shockley et al., 2004). The perception–production-link allows for the alignment to detailed pronunciation, due to traces stored in the lexicon that can later be retrieved, thereby increasing mutual intelligibility (Fowler & Galantucci, 2005; Liberman & Mattingly, 1985; Pickering & Garrod, 2004, 2013).

In sum, there are two mechanisms presumably responsible for speech accommodation—social motivation as a conscious voluntary mechanism and automatic adjustment as an unconscious and involuntary mechanism. The question is how these two mechanisms can be observed in non-native speech. In other words, provided that a desire of non-native speakers to achieve a high level of intelligibility can be assumed, does insufficient proficiency impede the application of accommodation strategies to affiliate with native speakers of their L2 and for automatisms to kick in?

### 1.2 Accommodation in L2

Foreign accented speech has been shown to be perceived as a social marker especially when compromising intelligibility (Dragojevic et al., 2016). It conveys, for instance, information about the proficiency level of the speakers and other social attributes associated with particular patterns of language use (Atagi & Bent, 2015; Giles & Ogay, 2007; Gluszek & Dovidio, 2010). Furthermore, speech of non-native speakers is often associated with negative attitudes (McKirnan & Hamayan, 1984), and their speakers are rated to have a lower social status and lower general competence (Nelson et al., 2016). Non-native speakers may be sensitive to such valuations and hence desire to adapt their verbal behavior in order to comply with attitudes and aspects of social identity (Giles & Johnson, 1987; Zuengler, 1991).

#### 1.2.1 Accommodation on the segmental level of L2 speech

Several factors have been investigated in order to account for accommodation effects or the lack thereof in non-native speech considering cognitive and perceptual signals. Recency is such a factor reported to influence accommodation in an imitation task by Rojczyk et al. (2013) studying burst release across word boundaries in Polish and English. The first plosive in two-word noun phrases with a stop sequence across the word boundary is not likely to be released in English but is released in Polish. Polish speakers of L2 English were found to adapt the non-release patterns of the English target language. The influence of distraction on accommodation has been addressed in an imitation task administered to Asian immigrants learning English as an L2 by Adamson and Regan (1991). The findings revealed that speakers do accommodate to the patterns of the model speakers but that the imitation is reduced when participants were distracted by an additional task prior to imitation. An additional factor found to affect accommodation is gender. Females were found to accommodate more than males but also both males and females accommodate more to males than to females (Bilous & Krauss, 1988; Willemyns et al., 1997).

Honorof et al. (2011) reported accommodation for the degree of velarization in imitations of a model speaker as quantified in the first formant–second formant distance. Furthermore, accommodation in the production of vowels has frequently been investigated (e.g., Beebe, 1981 for Chinese speakers of L2 Thai, and Rojczyk, 2013 for Polish speakers of L2 English). Burin and Ballier (2017), for instance, studied segmental vowel quality, vowel duration and speech rate. Their results for accommodation of vowel quality confirmed previous intralanguage (Babel, 2009) and interlanguage (Rojczyk, 2013) findings demonstrating a dependency of accommodation on vowel height. Low vowels are more likely to be accommodated than high vowels, presumably due to a more open oral cavity allowing for more articulatory freedom. Speech accommodation on the segmental level has moreover been observed as a result of novelty, atypicality, recency and immediacy for instance by Enzinna (2018), who also emphasizes the automatic nature of the process.

The individual phonetic and phonological phenomena studied so far are mostly observed in a controlled experimental setting. The current study, however, is based on interactions, namely conversations during the completion of a map task. Map tasks allow for a relatively high level of control of the speech material but at the same time for the collection of comparably natural speech.

#### 1.2.2 Accommodation of prosodic characteristics

Adaptation of suprasegmental characteristics of speech is an everyday phenomenon. When individuals talk to children or pets, for instance, they typically use higher pitch and a wider pitch range (Burnham et al., 2002; Fernald & Simon, 1984; Garnica, 1977). In a noisy environment, we speak with increased intensity (Black, 1949; Coulston et al., 2002; De Looze et al., 2011; Gregory & Hoyt, 1982; Lombard, 1911; Meltzer & Morris, 1971; Natale, 1975). This shows that suprasegmental characteristics can be changed depending on various factors such as the environment or the specific situation. The effects referred to as accommodation investigated in the present study, however, denote changes in an individual’s production that result in speech patterns that become more similar to those of a concrete and specific interlocutor in the course of an interaction. Both local and global aspects of pitch (or fundamental frequency (F0)) are found to be accommodated (De Looze et al., 2011; Gregory & Dagan, 1997; Gregory & Webster, 1996; Shepard et al., 2001). In addition, individuals reportedly accommodated temporal characteristics such as pausing and speech rate (De Looze et al., 2011; Edlund et al., 2009; Giles et al., 1991; Kousidis et al., 2009; Matarazzo et al., 1970; McGarva & Warner, 2009; ten Bosch et al., 2005).

However, findings about the features of spoken language, which are more or less likely to be subject to accommodation, are not conclusive. Snow (1995), for instance, found higher pitch, shorter sentences and a slower speech rate in conversations of native speakers with non-native speakers compared to conversations among native speakers. Smith (2007) reports the results of the analysis of an interactive task whereby native speakers of French give directions to both native and non-native speakers of French. Accommodation effects were found in a significantly greater pitch range in conversations with non-natives compared to those with French native interlocutors. However, speech rate and utterance duration were not modified in either setting. This contradicts Snow’s (1995) findings as well as those reported by Burin und Ballier (2017). They found speech rate adaptation in both an advanced learner of English and two British native speakers. Over the course of the interaction in English, the former was shown to increase speech rate, whereas the latter two slowed down. Such inconsistencies and ambiguities may be due to the above-mentioned competing mechanisms and factors involved.

Most studies on accommodation on the suprasegmental level of speech are concerned with global or gradient features such as F0 range, speech rate or pausing, that occur over the course of an interaction. There are exceptions though, for example, investigating local prosodic phenomena associated for instance with pitch accents or boundary tones operating below the global utterance level. Mennen (2004), for instance, investigated the gradient prosodic feature of peak alignment in categorically, that is, phonologically, identical rising prenuclear pitch patterns in Dutch and Greek. In this acquisition study, she found that the majority of native Dutch L2 learners of Greek were able to produce an adjusted L2 Greek pattern but that they failed to produce the alignment in the prenuclear pitch patterns like monolingual Dutch speakers. The results showed that it is possible to adjust gradient intonational features but that not all phonological contrasts within and across two languages can be maintained. Mennen’s (2004) study shows that apart from paralinguistic or extralinguistic functions, prosodic adaptation may also feature linguistic functions. Kaland (2014) confirmed this in a series of experiments on adaptation of contrastive intonation patterns in healthy and autistic subjects as well as in a cross-linguistic study with native speakers of Dutch and Italian. On the one hand, he demonstrates that contrastive intonation depends on a speaker’s knowledge and her assumption about the information she may share with the interlocutor. On the other hand, the author found that speaker pairs, adapting to each other but still maintaining a coherent way of producing contrastive intonation, were perceived as better adaptors than those simply copying each other’s intonation patterns. Finally, he showed that speakers’ adaptation depends on the linguistic properties of their native language. More specifically, Dutch speakers were more flexible adaptors than Italians were, because Dutch allows pitch accents to shift depending on information–structure requirements whereas Italian does not.

Lee et al. (2018) investigated whether accommodation takes place for particular prosodic properties such as choice, strength and excursion of boundary tones as well as phrase-final lengthening. They also differentiated between an individual speech task and a cooperative task carried out with native speakers of American English. The investigated dyads obtained in a maze navigation task revealed a great amount of variation between and within individual speakers. More specifically, there were differences across the temporal and intonational measures indicating firstly that accommodation takes place (as well as divergence and maintenance/persistence). However, speakers differed in their attention to the individual prosodic means of their interlocutors. Secondly, Lee et al.’s results showed that temporal and intonational aspects are “cognitively executed independently of one another” and not “governed by a single abstract aspect of phrasal structure” (Lee et al., 2018, p. 21). The authors also found that the adjustments in most dyads were unilateral and that effects of accommodation were observed even after an interaction has concluded.

Such findings, however, are not unchallenged. Tooley et al. (2018) found evidence for accommodation of speech rate but not for intonational phrase boundary and pitch accents. The authors concluded that speech rate as a paralinguistic means is planned independently from intonational features in speech production. Intonational phase boundaries and pitch accents, however, convey linguistic information to the listener and may therefore be represented differently and in preparation of production and planned together with other types of linguistic representation, such as cues to syntax, semantics, and discourse focus.

Trofimovich (2016) and colleagues studied several aspects of alignment in L2 speech, including the realization of initial /h/ (Trofimovich & Kennedy, 2014) and word stress patterns (Trofimovich, et al., 2014) in English L2 and found accommodation across groups of speakers from various native languages. The perceptual relevance of these effects was tested in similarity ratings by native speakers of English revealing that accommodation enhances comprehension. Crucially, accommodation to non-target-like productions did not take place.

### 1.3 Hypotheses

In summary, research on accommodation cannot be readily transferred to the analyses of accommodation in interlanguage conversations. In non-native speech the flexibility and ability to accommodate most likely depends on the individual’s repertoire. The repertoire in turn depends on the proficiency level of the individual. Individuals with a higher proficiency level have a larger pool of variants to select from in the target grammar whereas individuals with lower proficiency levels have a comparably smaller pool to select from and are therefore forced to revert to their L1 variants. Furthermore, learner grammars develop under the influence of linguistic and extralinguistic factors. One of the most important factors is quality and quantity of input. In addition, attitudes and motivation significantly influence language development. These factors dynamically interact with other factors and so far, there has been no break-through as to how we can weight those factors in the individuals’ developmental paths. The present study adds another factor, namely proficiency, to account for variation to see whether inadequate or insufficient language skills impede accommodation.

Moreover, the present study complements existing studies on accommodation in L2 speech by looking at accommodation effects on both the segmental and the suprasegmental levels of speech in a collaborative communication task.

The following hypotheses for the present study are derived based on the reviewed literature:

H1: Accommodation takes place in conversation among Spanish learners of German and in conversations with a native speaker of German. The effects are demonstrable on both the segmental and the suprasegmental levels of speech.
H2: Accommodation effects will depend on the proficiency level of the L2 learners of German. The choice of L2 variants may be reduced in speakers with a lower proficiency level when compared to highly proficient speakers. Therefore, I expect to find different patterns of convergence towards L2 variants and the maintenance of L1 variants depending on the proficiency level of the participant.
H3: Accommodation effects will depend on the interlocutor. Non-native speakers accommodate to a native speaker and highly proficient non-native speakers but not to a non-native speaker with a lower proficiency level because the effect is socially motivated and due to a conscious desire to sound more native-like and less foreign accented.

## 2 Materials and methods

The data for the present study are from speakers of the standard varieties of Spanish and German, namely Castilian or Peninsular Spanish and Northern Standard German. Crucially for the present investigation, the two varieties differ both on the segmental and on the suprasegmental levels so that the two levels of speech can be investigated within an experimental design and with the same participants.

Both Spanish and German show a wide range of variation in the realization of /r/ and both exhibit this variation depending on phonotactic constraints. The Spanish rhotic is produced as an alveolar tap or a trill. In initial position and in association with the double letter spelling <rr> (*perro* vs. *pero*) it is realized more frequently as a trill /r/. In the coda as well as in initial and final consonant clusters it is realized as a tap /ɾ/ and frequently accompanied by a schwa epenthesis [ə] (Bradley, 2004, 2006; Bradley & Schmeiser, 2003; Willis & Pedrosa, 1998).

German /r/ has been shown to vary considerably also (Göschel, 1971; Ulbrich, 1973, 2002; Ulbrich, & Ulbrich, 1998, 2007). A general cross-varietal tendency is found in the vocalization of /r/ to [ɐ] in postvocalic position. As a single consonant or in onset clusters, rhotics are found to be realized as alveolar [r] and uvular [ʀ] trills predominantly in southern varieties as well as in Austrian and Swiss German. In the rest of Germany as well as in northern parts of Switzerland (Leemann et al., 2018), prevocalic rhotics are produced as uvular fricatives [ʁ].

There are also differences on the suprasegmental level between German and Spanish relevant for the present study. Spanish is assumed to be syllable-timed (Amador-Hernandez, 1986; Solé, 1991) whereas German is considered to be stress-timed. This has implications for speech rhythm, the realization of stress and unstressed syllables, and intonation. Spanish intonation is assumed to be smoother and steadier, hence more monotonous, compared to German (Hirschfeld, 1988). More specifically, pitch accents are produced more regularly in relation to each other and with a smaller pitch excursion. Furthermore, the varying number of syllables between accented syllables in German as a stress-timed language is responsible for less articulatory tension in the unstressed syllables resulting in more reductions and assimilations.

These differences allow for a more precise specification of hypothesis 1. When accommodation takes place, Spanish L2 speakers of German will:

H1.1 increase the number of uvular fricatives in prevocalic position
H1.2 produce a larger pitch range on accented syllables
H1.3 increase the number of reductions and assimilations.

### 2.1 Participants

Twelve female speakers of Castilian Spanish (aged 19–31; average 24) with no reported speech, language, or hearing problems participated in the experiment. The subjects were living in the area of Lake Constance in Baden-Württemberg and were recruited at the University and the adult education center of Constance. In order to create relatively homogenous groups, sociodemographic data were obtained (see below). In addition, participants had to provide proof for a C1 level to be allocated to a group of highly proficient speakers (HP) and a B2.1 level to be allocated to a group of speakers with a lower proficiency level (LP). The allocation was according to the guidelines and the assessment of the Common European Framework of Reference for Languages (Council of Europe, 2011). Recordings took place during the summer semester 2018. Subjects were not paid and their participation was voluntary. The HP and LP participants were paired up for a collaborative task with three female control speakers: one highly proficient control (HPc) and one control of lower proficiency (LPc) (both native speakers of Castilian Spanish) as well as one native speaker of German. The control speakers were matched in education and age to the participants of the two groups. The collaborative task took place in the target language German. The HPc was chosen because of her native-like pronunciation, notable in the illustration of the results in Figures 3–5 showing that the HPc’s production of all three phonetic characteristics, namely /r/ realization, pitch range and articulation rate, appears to be more German-like than the HP participants’ realizations.

### 2.2 Procedure

Prior to the collaborative task, participants had to fill in a questionnaire to obtain sociodemographic data about their linguistic upbringing including the language background of their parents, about patterns of language use for both the L1 and the L2. Furthermore, details about any additional language(s), the time frame of acquisition such as age of acquisition, length of residence, attitudes towards German and stereotypes about German native speakers as well as facts about motivations to acquire German were obtained. Speech data were recorded during a collaborative map task in German, where a participant takes the role of a tourist and a control speaker that of a guide or vice versa. Map tasks allow for the comparison between languages, a relatively high level of control of the segmental and the suprasegmental level of speech and the collection of largely comparable natural speech while keeping the task the same. All three control speakers completed the task with each of the 12 participants. This means that every participant completed three procedurally identical map tasks. The content of the maps, however, was different in the three versions. The maps of the tourists did not match exactly the maps of the guides in that some information was reserved for either of the interlocutors and both had to inquire about them according to a task list given to them prior to the map task. Tasks included: to find out what films are playing at the cinema; to inquire on the daily menu in restaurants; life music playing at bars; newly acquired animals in the zoo; recently opened shops in the mall; new breeds of plants in the market garden; among others. The task list was handed out in order to increase comparable material. Recordings took place in a quiet room. During the recordings, participants and controls sat facing each other with the respective map in front of them. A half-height divider separated them. Recordings were done via Plantronics HW251N SupraPlus Wideband Headsets directly onto MacBook Pro computers. The total duration of analyzed recordings was 132 minutes for the HP speakers. The average duration of the map task was 22 minutes; the shortest recording was 15 minutes, the longest 35 minutes. The total duration for the LP speakers was 171 minutes. The duration of their map task recordings was on average longer with 28.5 minutes—the shortest recording was 18 minutes, the longest 34 minutes.

### 2.3 Data treatment and analyses

For all three phenomena analyzed, tokens for the individual analyses were taken from the first 30% of the recordings and the last 30% of the recordings corresponding to rect1 as early parts of the conversation and rect2 as late parts of the conversation, respectively, in order to see whether accommodation takes place. In total 471 word-initial /r/ realizations were analyzed, 263 produced by the HP speakers and 208 produced by the LP speakers. For the analysis of pitch range in prenuclear pitch accents 1237 tokens (663 HP and 574 LP) were analyzed. The individual numbers per participant in the two subject groups, HP and LP, are detailed in Table 1.

**Table 1.** Number of analyzed word-initial /r/ sounds and number of pitch accents produced by the highly proficient speakers (HP) and speakers with a lower proficiency level (LP) in early (rect1) and late (rect2) parts of the recordings.

Speakers	HP				LP			
	/r/ realizations		pitch accents		/r/ realizations		Pitch accents	
	rect1	rect2	rect1	rect2	rect1	rect2	rect1	rect2
1	21	36	43	56	21	10	34	46
2	13	21	42	60	28	16	62	51
3	15	23	57	61	16	23	59	52
4	17	18	53	55	19	21	43	33
5	26	19	36	45	17	9	56	43
6	29	25	72	83	15	13	46	49
Total	121	142	303	360	116	92	300	274

For the analysis of articulation rate, 20 sentences (10 sentences at rect1 and 10 sentences at rect2) of approximately the same length were chosen from the data of each of the subjects as detailed in subsection 2.3.3.

#### 2.3.1 Word initial /r/

For the analysis of /r/ realization, all words with an initial /r/ produced by the subjects were extracted. They had to be produced in either nuclear or prenuclear accent position. Several targets were part of the map and the tasks (e.g., *Ringe*, *Riese*, *Reifen*, *Reise*, *Reibekuchen*, *Rotwurst*, *Rot*, *Rosen*, *Rosinen*, *Regal*, *Reh*, *Regen*, *Reben*, *Rat*, *Rad*, *Rabe*, *Rand*, *Rubin*, and *Rubens*).

The analysis was limited to accented words with /r/ in word-initial position, because these are expected to be realized both in German and in Spanish with a full consonantal /r/ variant. As detailed above, word initial /r/ is produced as a uvular fricative [ʁ] in German and as an alveolar trill [r] in Castilian Spanish. During an informal conversation with a native speaker of Castilian Spanish prior to the experiment, subjects were asked to confirm that Castilian Spanish was their spoken variety. Annotation and acoustic measurements were carried out using Praat (Boersma & Weenink, 2009). Target words were segmented by the author and annotated auditorily by the author and three native speakers of Spanish. Subsequently, /r/ realizations were extracted using a script and their annotation as either trill [r] or fricative [ʁ] were acoustically verified. A trill is characterized by two or more brief occlusions between the tongue apex and the alveolar ridge (Henriksen & Willis, 2010; Hualde, 2005, p. 181). The uvular fricative is characterized by a more homogenous fricative structure.

**Figure 1.**
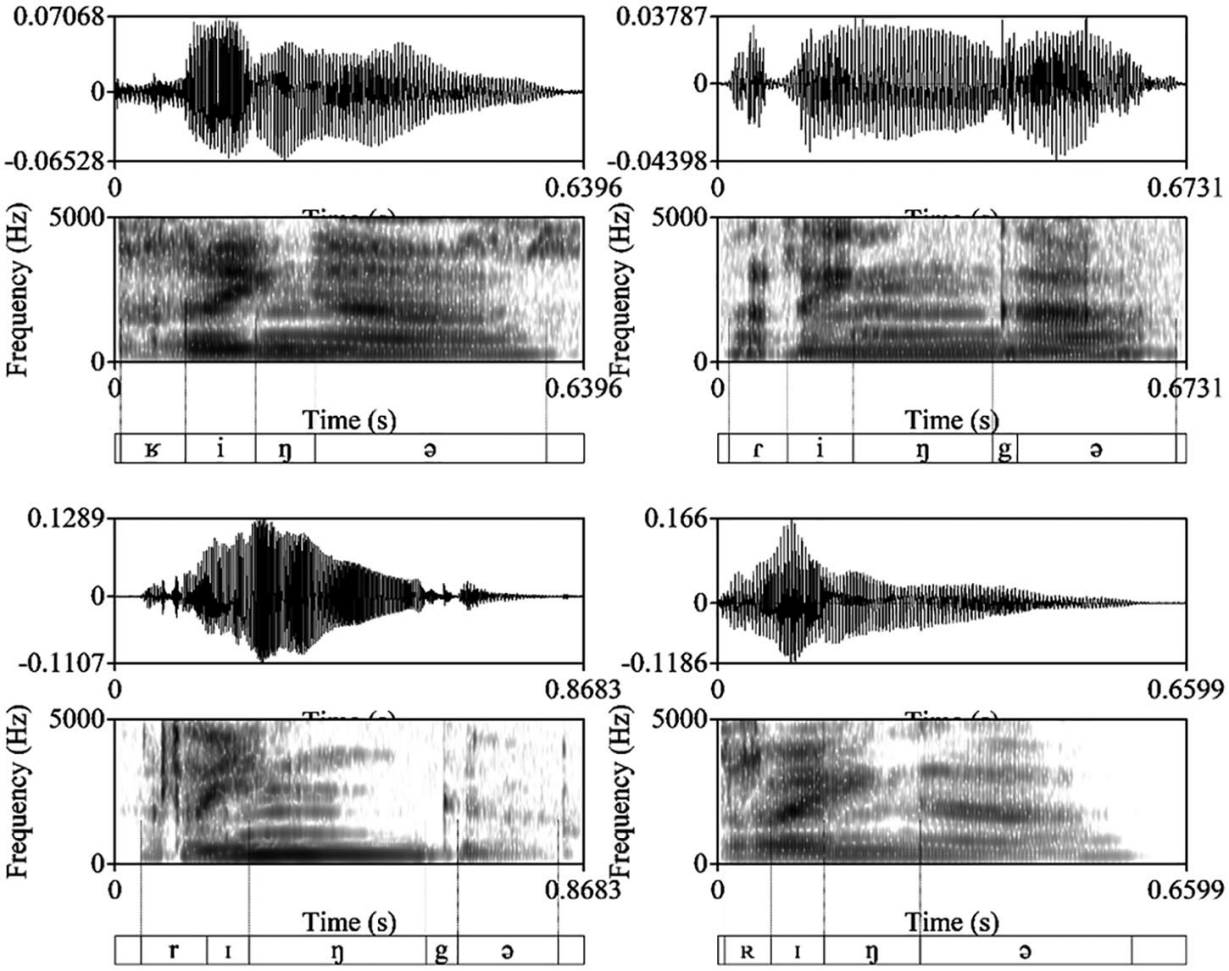
Examples of four types of /r/ realization found in the data in the word *Ringe*: top left [ʁɪŋə] with an initial uvular fricative; top right [ɾiŋgə] with an initial flap; bottom left [rɪŋgə] with an initial apical trill; and bottom right [ʀɪŋə] with a uvular trill.

A generally well-marked characteristic of the [ʁ] is the approximation of the third and the fourth formant in the center of the fricative at around 3000–3500 Hz. There were some more variants in the data such as trill realizations in uvular position, sometimes with relatively large portions of friction, and taps. The types of /r/ realizations are exemplified in Figure 1(a–d).

#### 2.3.2 Pitch range on prenuclear accents

For the analysis of pitch range, prominent syllables were identified in declarative broad-focus sentences by the author and three native speakers of German. Only initially accented bisyllabic words with sonorant material in prenuclear accent position were used. This is because nuclear accents in utterance final position are realized with a sharp utterance final fall in declarative sentences often accompanied by creaky voice quality. Creaky voice is acoustically characterized by irregular glottal cycles and a longer closed phase compared to modal voice (Hollien et al., 1977). Perceptually, the voice quality is perceived as a comparably sudden decrease in pitch (Kreiman, 1982). Since this voice quality has been shown to undergo accommodation effects, their occurrences were excluded from the analysis (Pillot-Loiseau et al., 2019). In total 1237 prenuclear pitch accents were analyzed, 663 produced by the HP speakers and 574 produced by the LP speakers. The individual numbers per participants are detailed in Table 1.

**Figure 2.**
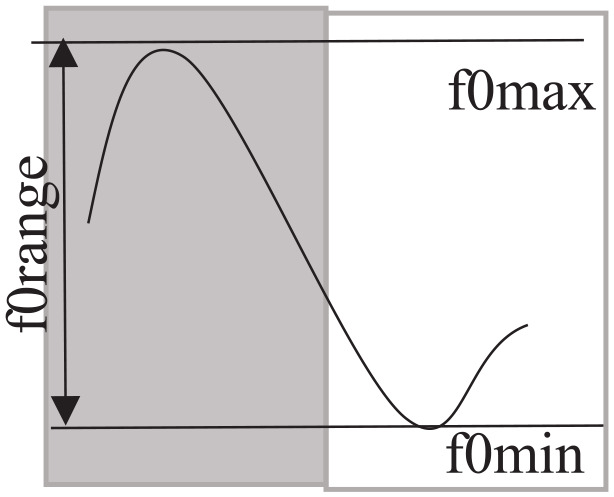
Illustration of measurements obtained for the calculation of the pitch range (forange) with the maximum fundamental frequency (fomax), the minimum fundamental frequency (fomin). The gray rectangle illustrates the accented syllable.

Since the automatic F0 extraction of extreme values can be unreliable, measurements were taken manually in Pratt (Boersma & Weenink, 2009) at the highest point of the fundamental frequency (F0max), reached within the accented syllable of the target word (or at the beginning of the subsequent unaccented syllable), and at the end of the immediately following fall in fundamental frequency (F0min) as illustrated in Figure 2. The pitch range (F0range) was calculated by subtracting F0min from F0max. In order to allow for a comparison between individuals, obtained values were converted to semitones.

#### 2.3.3 Speech rate and articulation rate

Speech rate and articulation rate are affected by segmental and suprasegmental characteristics of speech. Speech rate includes pauses and is defined as the number of production units per time unit, mostly syllables per second (Crystal & House, 1990). Articulation rate is not easily defined. Several measures have previously been evaluated (Trouvain et al., 2001). In order to address accommodation in the present data, two types of articulation rate were distinguished: (a) the intended articulation rate, which is based on the canonical underlying phones, assumed to be stored in the mental lexicon; and (b) the actually realized articulation rate. This rate refers to acoustically measured phones excluding not only pauses but also qualitative and quantitative reductions and deletions (Koreman, 2006). In the present study, only deletions of a complete phone segment were considered, because “[I]t is expected that realizations with more numerous deletions also show a greater number of incomplete reductions, since these can be considered as a less extreme but otherwise similar effect of a sloppy articulation” (Koreman, 2016, p. 586). This is relevant for the present investigation since, as mentioned above, native speakers of Spanish are assumed to reduce unaccented syllables between accented syllables less frequently compared to native speakers of German because of their different L1 rhythm patterns (Hirschfeld, 1988). This means that the ratio of realized to intended articulation rate (i.e., ARr/ARi) should be higher in Castilian Spanish L2 learners of German when compared to the German control. For the analysis, 20 sentences (10 sentences at rect1 and 10 sentences at rect2) of approximately the same length were chosen from the data of each of the subjects. In the selected sentences, there were no repetitions or other obvious interruptions that occurred, for example, due to the lack of vocabulary or difficulties with the map. The number of intended phones was obtained from a phonemic transcription of the sentences, and the number of realized phones from their broad phonetic transcription.

### 2.4 Statistical analysis

Statistical analyses were carried out using R (R Core Team, 2013) and the lme4 package (Bates et al., 2015). For the analysis of the categorical variable /r/ realization, a generalized linear mixed model analysis was carried out. The regression analysis includes both fixed effects (intended manipulation built into the experimental design) and random effects (not manipulated or experimentally controlled sources of variability) (Baayen et al., 2008). The fixed effects in the present study are early and late *recording time rect* (rect1 and rect2, reference level rect1), *proficiency level PL* (HP vs. LP, reference level HP) and *interlocutor IL* (G vs. LPc vs. HPc, reference level G). Random slopes for the fixed effects were added to the random-effects structure and kept if this improved the fit of the model (Matuschek et al., 2017). A comparison of the model’s log-likelihood using the R-function anova() was employed to achieve the best-fitted model.

For the statistical analyses of the phonetic variables pitch range and articulation rate, the dependent variables were analyzed using linear mixed-effects regression models with the same specification and procedure for fitting the model as employed in the analysis for the categorical data. Satterthwaite approximation implemented in the R-library lmerTest (Kuznetsova et al., 2016) was used to estimate the degrees of freedom (df) and to obtain *p* values. *p* values were adjusted using the Benjamini–Hochberg correction (Benjamini & Hochberg, 1995) since multiple variables were tested. Both the raw and the adjusted *p* values are reported below.

## 3 Results

### 3.1 Realization of /r/

A total of 417 tokens of prevocalic /r/ were statistically analyzed according to three predictors: recording time *rect*; proficiency level *PL*; and interlocutor *IL*. Random intercepts were allowed for experimental *subjects*. The results are summarized in Table 2.

**Table 2.** Results of a generalized linear mixed-effects model for target [ʁ] realization.

		rect_1	rect_2	Estimate	Standard error	*z* ratio	*p* value
Highly proficient speakers	Reference level (G)	43	55	5.76	1.06	5.42	<0.0001
	Highly proficient control (HPc)	37	47	3.58	0.92	2.8	=0.00515
	Control for low proficiency (LPc)	35	32	−1.44	0.08	−1.07	>0.5
Speakers with a lower proficiency level	G	32	28	−1.01	0.03	−0.95	>0.8
	HPc	22	19	−1.21	0.04	−0.78	>0.1
	LPc	21	23	1.09	0.07	1.56	>0.2

A predictor of recording time was not significant on its own (*p* = > 0.9), but a predictor for proficiency level, ß = -1.72, standard error (*SE*) = 0.64, *z* = -2.63, *p* = *p_adjusted_* = 0.0381*, and interlocutor, ß = -3.33, *SE* = 1.078, z = -2.09, *p* = *p_adjusted_* = 0.0201*, was. Furthermore, the three predictors showed a significant interaction with each other to affect the likelihood of native-like realization of /r/ as a uvular fricative as detailed below.

**Figure 3.**
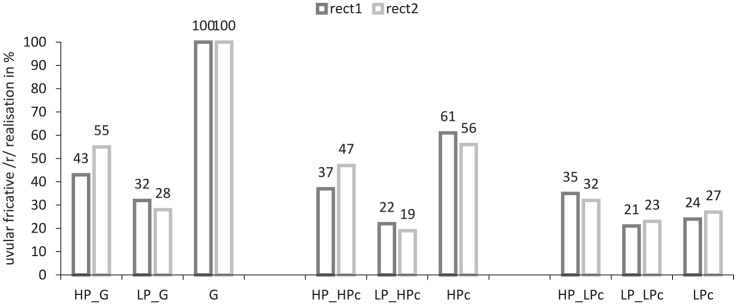
Native-like /r/ realization as uvular fricative in word initial position at recording time 1 (rect1) and recording time 2 (rect2) highly proficient (HP) speakers and speakers with a lower proficiency level (LP) in the map-task completion with the native German control (G), with the highly proficient control (HPc), and the control for low proficiency (LPc).

The number of native-like realizations of uvular fricative is plotted in Figure 3. The realizations of the three control speakers appeared to be relatively stable throughout the completion of the map tasks. Note that their data are averaged across all 12 conversations held with six HP and six LP speakers. The German control produced only target-like /r/ realizations (100%), whereas the HPc produced about half of initial /r/ as a uvular fricative (61% at rect1 and 56% at rect2). Approximately a third (24% at rect1 and 27% at rect2) of the target words were produced with a uvular fricative by the control of low proficiency. Considering the subject groups, the results, illustrated in Figure 3, show that HP speakers’ target-like realizations increased during the conversation with both the native German control (increase by 8 percentage points; ß = 5.76, *SE* = 1.06, *z* = 5.416, *p* = *p_adjusted_* =⩽ 0.0001) and the HPc (increase by 10 percentage points; ß = 3.58, *SE* = 0.92, *z* = 2.798, *p* = *p_adjusted_* = 0.00515) indicating convergence towards the realization of the uvular fricative. Overall, HP speakers produced significantly fewer uvular fricatives during the completion of the task with the control of low proficiency, ß = -1.6567, *SE* = 0.9181, *z* = -1.804, *p* = 0,0021, *p_adjusted_* = 0.0116, and even less at rect2. However, the difference of three percentage points between /r/ realizations at rect1 (35%) and rect2 (32%) of HP speakers in conversation with the control of low proficiency was not significant, *p* = *p_adjusted_* > 0.5. In the realizations of LP speakers, no accommodation effect can be observed. The already relatively low proportion of native-like realizations even decreases slightly during the map task completed with the German control (by four percentage points) and the HPc (by three percentage points). The difference, however, is not significant, *p* = *p_adjusted_* > 0.8. The interaction of the three variables suggests that the effect of one predictor variable depended on the value of other predictors. The effect of these three interacting factors is plotted in Figure 3.

### 3.2 Pitch range

A total of 1237 tokens (663 HP and 574 LP) was analyzed. To model pitch range data, a linear mixed-mixed effects model (LMM) was implemented with the lmr function from lme4 and lmerTest packages in R. Satterthwaite approximation implemented in the R-library lmerTest (Kuznetsova et al., 2016) was used to estimate the *df* and to obtain *p* values. Proficiency level (HP and LP), interlocutor (G, HPc, and LPc) and recording time (rect1 and rect2) were added to the model as fixed predictors, together with their three-way interaction. Random intercepts were allowed for experimental *subjects*. The results of the statistical analysis are summarized in Table 3.

**Table 3.** Results of a linear mixed-effects model for pitch range.

		rect_1	rect_2	Estimate	Standard error	Degrees of freedom	*t* ratio	*p* value
Highly proficient speakers	Reference level (G)	3.5	4.6	1.9	0.46	37.9	2.02	<0.0016
	Highly proficient control (HPc)	3.1	4.2	2.3	0.51	35.1	2.36	<0.031
	Control for low proficiency (LPc)	3.4	3.7	1.1	0.67	34.2	1.21	>0.8
Speakers with a lower proficiency level	G	3.1	3.2	0.27	0.01	37.2	1.01	>0.1
	HPc	2.8	3.4	1.2	0.21	31.9	2.001	<0.011
	LPc	2.6	2.9	1.7	0.14	41.2	2.191	<0.019

**Figure 4.**
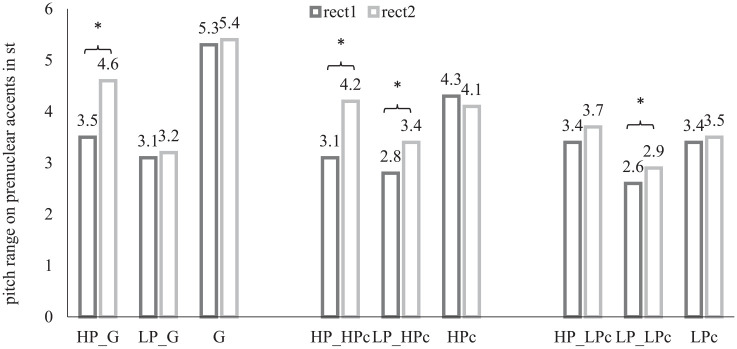
Pitch range in phoneme level for prenuclear accents at recording time 1 (rect1) and recording time 2 (rect2) highly proficient (HP) speakers and speakers with a lower proficiency level (LP) in the map-task completion with the native German control (G), with the highly proficient control (HPc), and the control for low proficiency (LPc).

Pitch range was measured in Hz on accented syllables in prenuclear position of bisyllabic words as illustrated in Figure 2. The obtained Hz values were converted into semitones prior to statistical analysis. The results for pitch range, illustrated in Figure 4, show that pitch range is generally larger at rect2 compared to rect1, ß = 1.7, *SE* = 0.31, *df* = 31,1, *t* = 2.512, *p* = *p*_adjusted_ < 0.0062, except for the HPc (4.3 semi tones (st) at rect1 and 4.1 st at rect2). Note again, that the data of the controls are pooled across all 12 conversations with the six HP and the six LP speakers. The German control produces the largest pitch range, G: 5.3 st at rect1 and 5.4 st at rect2, the HPc’s pitch range is about 1 st smaller, HPc: 4.3 st at rect1 and 4.1 st at rect2, and the pitch range of the control of low proficiency is about 2 st smaller compared to the German control, LPc: 3.4 st at rect1 and 3.5 st at rect2.

Considering the two subject groups, HP speakers produce a larger pitch range compared to the LP speakers, ß = -2.3, *SE* = 0.72, *df* = 28,2, *t* = -2.354, *p* = *p*_adjusted_ < 0.0093. Comparing the data of the two recording times, HP speakers’ pitch range converges towards the larger pitch range during the collaborative task with both the German control, 3.5 st at rect1 vs. 4.6 st at rect2, ß = 1.9, *SE* = 0.46, *df* = 37.9, *t* = 2.0213, *p* = *p*_adjusted_ < 0.0016, and the HPc, 3.1st at rect1 vs. 4.2 st at rect2, ß = 2.3, *SE* = 0.51, *df* = 35,1, *t* = 2.362, *p* = *p*_adjusted_ < 0.031. The increased pitch range by 0.3 st in the conversation with the control of low proficiency is not significant, 3.4 st at rect1 vs. 3.7 st at rect2, *p* = *p*_adjusted_ > 0.8.

Pitch range in the speech samples obtained from LP speakers also increases by 0.6 st, but only in the map task completed with the HPc, 2.8 st at rect1 vs. 3.4 st at rect2, ß = 1.2, *SE* = 0.21, *df* = 31,9, *t* = 2.001, *p* = *p*_adjusted_ < 0.011, and the control of low proficiency, 2.6 st at rect1 vs 2.9 st at rect2, ß = 1.7, *SE* = 0.14, *df* = 41,2, *t* = 2.191, *p* = *p*_adjusted_ < 0.019, but not in the conversation with the German native control. This indicates that accommodation in pitch range only occurs in conversations with non-native controls even though LP speakers produce the largest pitch range both at rect1 and rect2, 3.1 st at rect1 vs 3.2 st at rect2, *p* = *p*_adjusted_ < 0.1, in conversations with the German control.

### 3.3 Articulation rate

**Figure 5.**
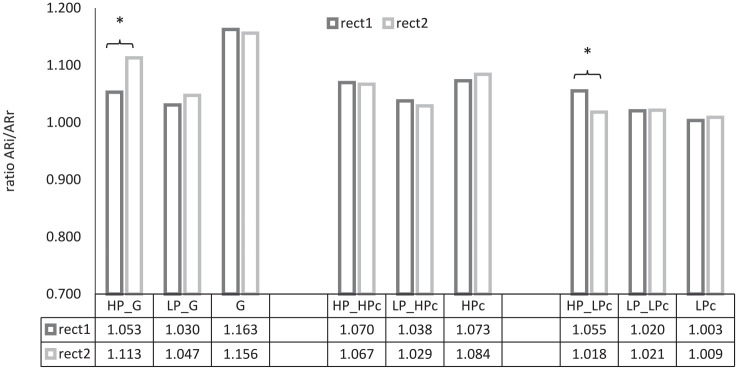
Ratio between intended (ARi) and realized articulation rate (ARr) at recording time 1 (rect1) and recording time 2 (rect2) of highly proficient (HP) speakers and speakers with a lower proficiency level (LP) in the map-task completion with the native German control (G), with the highly proficient control (HPc), and the control for low proficiency (LPc).

The experimental setting for data collection was used to ensure a relatively spontaneous interaction between the participants in the completion of the collaborative task. Collecting spontaneous data frequently goes at the expense of fluency, a measure highly related to speech rate, which includes hesitations, pausing, repetitions, etc. In order to control for those and to exclude such phenomena, the comparison was based on two types of articulation rate, namely a measure of articulation rate based on the number of phones that speakers intended to produce (ARi) and an articulation rate based on the actually realized phones (ARr). The analysis for all speakers was based on 10 sentences each of comparable length during the initial 30% (i.e., rect1) and the final 30% (i.e., rect2) of the collaborative tasks. Articulation rate was submitted to the statistical analysis as a ratio of intended and realized articulation rate, ARi/ARr. Figure 5 illustrates the results for the comparison of the ratio between intended and realized phones.

To model articulation rate data, a LMM was implemented with the same specification as described for pitch range data (see subsection 3.2). The results of the analysis for articulation rate are summarized in Table 4.

**Table 4.** Results of a linear mixed-effects model for articulation rate.

		rect_1	rect_2	Estimate	Standard error	Degrees of freedom	*t* ratio	*p* value
Highly proficient speakers	Reference level (G)	1.05	1.11	0.0072	0.0046	26.3	2.191	<0.019
	Highly proficient control (HPc)	1.07	1.067	−0.0032	0.001	23.5	−1.17	>0.8
	Control for low proficiency (LPc)	1.05	1.01	−0.0056	0.0014	18.2	−2.242	<0.069
Speakers with a lower proficiency level	G	1.03	1.05	0.0027	0.0085	21.9	1.91	>0.1
	HPc	1.04	1.03	−0.0024	0.0042	27.1	−1.29	>0.1
	LPc	1.02	1.02	0.0012	0.0006	28.2	0.93	>0.5

The difference between intended and realized articulation rate is largest in the data of the German control and smallest in the data of the control of low proficiency, G = 1.16; HPc = 1.08; LPc = 1.006, indicating that most reductions were found in the German control’s data and the fewest in the data of the control of low proficiency. Significant effects were only found in the data of HP speakers in conversations with the German control and the control of low proficiency. The ratio of intended and realized phones increases in the conversation with the German control, 1.05 at rect1 vs. 1.11 at rect2, ß = 0.0072, *SE* = 0.0046, *df* = 26,3, *t* = 2.191, *p* = *p*_adjusted_ < 0.019; however, it decreases in the completion of the task with the control of low proficiency, 1.05 at rect1 vs 1.01 at rect2, ß = -0.0056, *SE* = 0.0014, *df* = 18,2, *t* = -2.242, *p* = *p*_adjusted_ < 0.069.

The averaged ratio of ARi/ARr obtained from the group of HP speakers does not differ from that of the HPc, *p* = 0.1; *p*_adjusted_ > 0.12—this is most likely due to the fact that the HPc’s ratio does not differ from the average of the HP speakers so that it possibly does not trigger accommodation. LP speakers do not converge towards any of the three controls (with the German control: 1.04; with the HPc: 1.03, with the control of low proficiency: 1.02; *p* = 0.87; *p*_adjusted_ > 0.91). Their ratio is overall smaller compared to that of the HP speakers, indicating fewer phone reductions in the speech sample, LP 1.03 vs HP 1.06, ß = 0.0056, *SE* = 0.0014, *df* = 22,1, *t* = 2.242, *p* = *p*_adjusted_ < 0.069.

## 4 Discussion

The present study experimentally investigated whether non-native speakers accommodate towards native speakers in a conversational task. In addition, the role of the interlocutor and the proficiency level were addressed. The main findings are summarized below.

### 4.1 Accommodation of both segmental and suprasegmental characteristics of speech

(a) Spanish L2 learners of German were found to accommodate to native speakers of German in the more target-like realizations of /r/ and pitch excursion on prenuclear accented syllables as well as in articulation rate.

### 4.2 Influence of proficiency level

(b) Accommodation was found in the data of highly proficient learners for all three phenomena investigated. Learners with a lower proficiency level only accommodated on pitch range excursion on prenuclear accented syllables.

### 4.3 Influence of the proficiency level of the interlocutor

(c) Highly proficient Spanish L2 learners of German accommodated to both the HPc and the native control in the realization of /r/ and the pitch excursion on prenuclear accented syllables. Furthermore, highly proficient leaners accommodated their articulation rate to all controls, that is, independently of the proficiency level.
(d) Learners with a lower proficiency level only accommodated to the HPc in the realization of pitch range excursion.

In the following, the results will be evaluated in light of the hypotheses expounded in subsection 1.3. In hypothesis H1, accommodation effects were predicted for both segmental and suprasegmental characteristics of speech. The hypothesis was confirmed by the results. Non-native speakers of L2 German were found to increase the number of target-like /r/ realizations, the pitch range excursion and the number of reductions with the exception of highly proficient speakers in conversations with the control of low proficiency. Here, a decrease of reductions was found. Overall, the effects were more pronounced in the realization of /r/ and the realization of pitch range excursion than in the articulation rate as quantified in terms of number of reductions.

The effects differed between the two groups of high and low proficiency levels as predicted by hypothesis H2. Highly proficient speakers of L2 German showed more accommodation compared to the group of participants with a lower proficiency level. The latter were only found to accommodate to non-native speakers of German but not to the German interlocutor. Hypothesis H3, stating that accommodation will not take place in conversations with speakers of a low proficiency level, could not be confirmed. Accommodation was not only found towards native speakers of German but also towards non-native speakers of German. Interestingly, speakers of a higher proficiency level also adjusted to non-native speakers with a lower proficiency level. The effect, however, was only found in the articulation rate (ARi/ARr). Highly proficient speakers produced fewer reductions, hence they articulated more carefully when speaking with an interlocutor of a lower proficiency level. This observation is likely to be the result of a conscious effort to make the speech more intelligible and thereby to facilitate the communication (Pickering & Garrod, 2006; Shepard et al., 2001), an effect previously found in conversations of native speakers with non-native speakers (Gass & Varonis, 1984). However, as in the case of the present study, non-native speakers have also been found to adopt various factors of their speech in order to increase intelligibility for other non-native interlocutors in support of a fluent conversation (Davis, 2003; Ellis, 1985; Smith, 2007). This implies that increasing or decreasing the number of reductions is a conscious process and can strategically be employed by a speaker (Giles et al., 1991).

The motives of the highly proficient L2 speakers in the present study appear to be both, affective (Tajfel & Turner, 1986) and cognitive (Thakerar et al., 1982). Increasing the number of reductions between utterance-level stressed syllables makes the speech more native-German like, hence more stress-timed. This is in line with a presumed desire of non-native speakers, voluntarily acquiring a second or an additional language, to reduce a foreign accent in order to sound more native-like. Decreasing the number of reductions at the expense of nativeness in the target language, on the other hand, makes the speech more intelligible for less experienced L2 speakers of German. Accommodation to an interlocutor with a lower proficiency level thus indicates that participants in the current experiment consider intelligibility as more important than nativeness when interacting with an interlocutor of a lower proficiency level.

Alignment to a speaker with a lower proficiency level, however, can also be due to a similarity affiliation effect (Enzinna, 2018). Both linguistic background and the fact that the Spanish speakers are outsiders in relation to the native German speech community may trigger social affiliation to a linguistically and socially similar outsider group.

However, accommodation effects similar to that in the articulation rate are not found in the analysis of /r/ realizations and pitch range excursion because highly proficient speakers adjusted only to the native German and the HPc. A possible reason may be that the contribution to foreign accentedness varies between the three phenomena (Ulbrich & Mennen, 2016). Trill realizations and a smaller pitch range on accented prenuclear syllables may be perceived as stronger cues for foreign accentedness and at the same time impede intelligibility to a lesser extent than reductions so that gauging motives allow for the prevalence of affective over cognitive motives.

Considering the participants with a lower proficiency level, the findings of the present study reveal accommodation only towards the HPc but not towards the native speaker of German. This could be an effect of familiarity and L2 demands on the production–perception link. Due to their familiarity with the non-native Spanish accent in the German target language, the group of speakers with a lower proficiency level may overall perceive the speech of the HPc as less different from their own speech. It may therefore become easier for them to notice some aspects to accommodate to, especially when these aspects do not involve additional articulatory resources or motoric skills. This could explain the fact that speakers with a lower proficiency level were found to adjust the pitch range excursion but not the uvular fricative realization of /r/. The realization of a larger pitch range is based on more subglottal pressure and increased articulatory tension whereas the fricative realization [ʁ] implies both a different manner and a different place of articulation (and at least partly also the change from a mostly voiced trill to voiceless fricative [χ]). Therefore, participants are likely to perceive the difference between the alveolar trill and the uvular fricative but may not be able to articulatorily implement the difference due to a lack of practice so that motor planning cannot take place according to perception (Chartrand & Bargh, 1999; Dijksterhuis & Bargh, 2001).

This implies that both similarity affiliation and perceptual fluency (Reber et al., 2004) may account for the observed differences in accommodation between the phenomena investigated and between the two groups of speakers. More target-like productions of the HPc are possibly more accessible to the speakers of low proficiency because of their familiarity with the accent. In addition, the alignment to the native speaker may involve extra demands that L2 production imposes on the L2 speakers, and hence block possible alignment (Kim & Bradlow, 2011).

A necessary follow up would involve a test on how perceptually salient the variables investigated in the present study are for the identification of a non-native accent and thus for the association of social features to speakers using these variables, as previously done for regional dialects and ethnicity by Graff et al. (1986) and Torbert (2004).

Perceptual salience may be indirectly mirrored in accommodation towards native and non-native speakers. The more salient the differences are between acoustic phonetic cues of the target language in the realization of individual sounds, the more likely they are perceived as meaningful markers and hence trigger accommodation. It seems natural that salience is determined by the acoustic space and patterns of language use and that non-native speakers will attempt the greatest impact of accommodation by trying to overcome the most salient differences between their own and the target language. However, this may be prohibited by insufficient articulatory skills. The question whether such effects are not only the result of conscious strategic manipulations but also due to automatisms still has to be answered.

The results of the present study provide evidence for cross-linguistic influence and thus show that linguistic representation can dynamically change, but that the change at least partly depends on the proficiency level and the interlocutor. These findings should be taken into consideration in the design and administration of language instruction in linguistic research.

## 5 Conclusion

This paper showed that both segmental and suprasegmental characteristics are accommodated by non-native speakers of German in conversation with both native and non-native interlocutors. The findings reveal that experienced speakers in the study do show substantial amounts of accommodation in conversation with native and non-native interlocutors, but also that the effects are influenced by social affiliation and maintaining intelligibility. There are still open questions in the research program investigating accommodation of non-native speakers to native speakers of a language. The role of the level of proficiency, the temporal extent to which accommodated characteristics can be maintained as well as the influence of perceptual salience of and the familiarity with specific phonetic and/or phonological characteristics of speech, need to be further explored. In the case of the segmental and suprasegmental characteristics, both perceptual salience and familiarity appear to be necessary requirements met only with the achievement of a high proficiency level.
